# Endoscopic hand suturing of a covered self-expandable metal stent to prevent migration in malignant gastric outlet obstruction

**DOI:** 10.1055/a-2313-3869

**Published:** 2024-05-17

**Authors:** Daijiro Shiomi, Kazuya Sumi, Yuki Kawasaki, Norihiro Nomura, Jun Ushio, Takayoshi Ito, Haruhiro Inoue

**Affiliations:** 1 378609 Digestive Diseases Center, Showa University Koto Toyosu Hospital, Koto-ku, Japan

Self-expandable metal stents (SEMSs) have been shown to be safe and effective palliative treatments for malignant gastric outlet obstruction (GOO) 1 . Both covered SEMSs (CSEMSs) and uncovered SEMSs (USEMSs) have been used, with no observable differences in clinical outcomes demonstrated; however, tumor in-growth is a concern with USEMSs, whereas migration is an issue for CSEMSs 2 . We used an endoscopic hand-suturing device to fix a CSEMS that was placed to prevent bleeding from malignant GOO, to avoid stent migration.

An 85-year-old woman presented with anemia and malignant GOO caused by bleeding from a duodenal cancerous mass ( Fig. 1 ). Aggressive treatment was not appropriate because of her poor performance status. We therefore performed palliative treatment using a CSEMS (HANAROSTENT Naturfit Flare duodenum/pylorus stent; Boston Scientific, Marlborough, Massachusetts, USA) to prevent bleeding and relieve the stenosis. Because the stenosis of the duodenum was mild, we were concerned about possible stent migration. The uncovered proximal flare remained in the pyloric canal ( Fig. 2 ) and was sutured using an endoscopic hand-suturing device (SutuArt; Olympus Medical Systems, Tokyo, Japan) and a suture needle with barbed thread (V-Loc; Medtronic, Dublin, Ireland), which can be fixed simply by pulling the thread, without any need for knotting, owing to the barbs ( Fig. 3 ; Video 1 ).

**Fig. 1:**
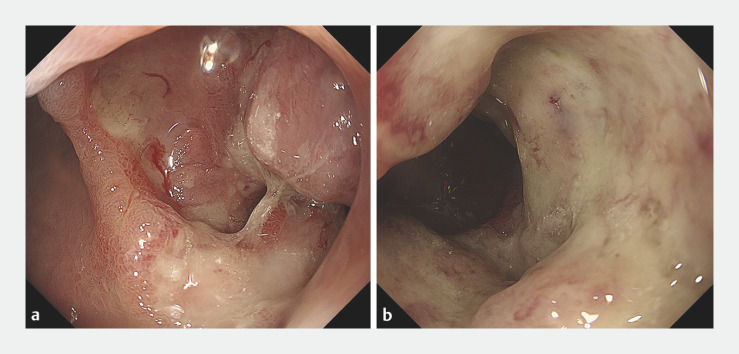
Endoscopic images showing stenosis and bleeding caused by a duodenal cancerous mass.

**Fig. 2:**
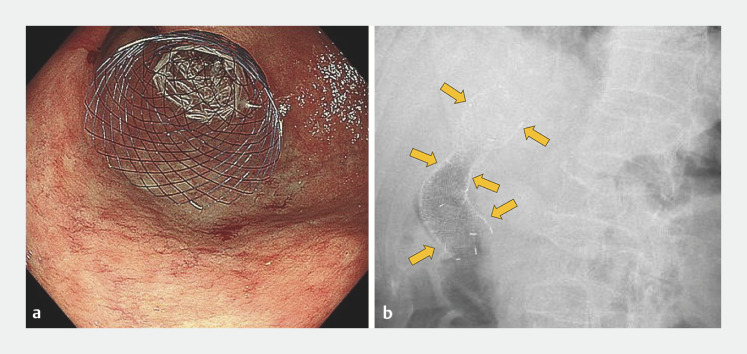
A duodenal covered self-expandable metal stent placed with the uncovered proximal flare remaining in the pyloric canal is seen on: a endoscopic view; b radiographic imaging.

**Fig. 3:**
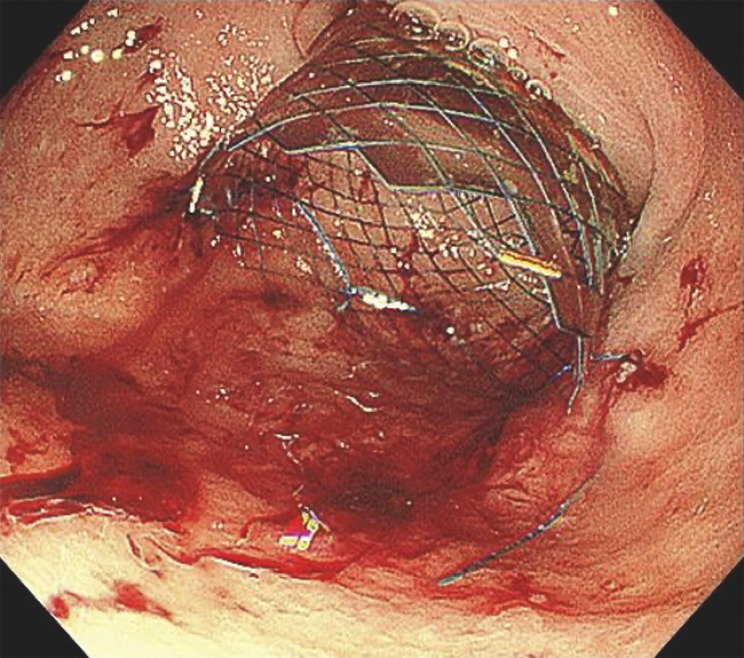
Endoscopic image showing the duodenal stent after fixation using the endoscopic hand-suturing device.

Endoscopic hand suturing was used to fix a covered self-expanding metal stent that was being placed as a palliative treatment to prevent bleeding and consequent anemia in a patient with malignant gastric outlet obstruction.Video 1

The procedure was completed without any major adverse events, and radiographic imaging 2 weeks later detected no evidence of stent migration ( Fig. 4 ). The patient had been requiring blood transfusions before the procedure, but her anemia was substantially improved postoperatively, with transfusions no longer required.

**Fig. 4:**
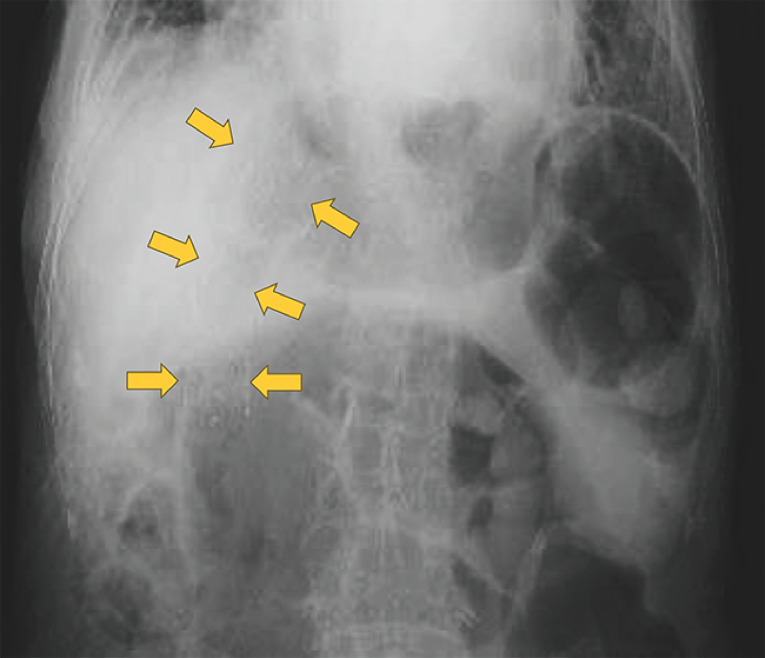
Radiographic image taken 2 weeks later showing no evidence of stent migration.

The fixation of SEMSs to the digestive tract has been reported using various clips 3 4 . Our case demonstrates that endoscopic hand suturing with SutuArt and V-Loc is simple, with the fixation being easy to remove by cutting the thread. This approach might be useful for SEMS fixation in the palliative treatment of malignant GOO.

Endoscopy_UCTN_Code_TTT_1AO_2AZ

## Notes

Endoscopy E-Videos https://eref.thieme.de/e-videos
*E-Videos* is an open access online section of the journal *Endoscopy* , reporting on interesting cases and new techniques in gastroenterological endoscopy. All papers include a high-quality video and are published with a Creative Commons CC-BY license. Endoscopy E-Videos qualify for HINARI discounts and waivers and eligibility is automatically checked during the submission process. We grant 100% waivers to articles whose corresponding authors are based in Group A countries and 50% waivers to those who are based in Group B countries as classified by Research4Life (see: https://www.research4life.org/access/eligibility/ ). This section has its own submission website at https://mc.manuscriptcentral.com/e-videos .
